# Blood Culture Contamination: A Single General Hospital Experience of 2-Year Retrospective Study

**DOI:** 10.3390/ijerph19053009

**Published:** 2022-03-04

**Authors:** Anna Tenderenda, Monika Łysakowska, Robert Dargiewicz, Anna Gawron-Skarbek

**Affiliations:** 1 Department of Geriatrics, Medical University of Lodz, 90-647 Lodz, Poland; 2 Department of Microbiology and Medical Laboratory Immunology, Medical University of Lodz, 90-213 Lodz, Poland; monika.lysakowska@umed.lodz.pl; 3 Department of Physiotherapy, Academy of Medical Science in Bialystok, 15-875 Bialystok, Poland; e@kiero.net

**Keywords:** blood culture contamination, bloodstream infections, coagulase-negative staphylococci

## Abstract

In the event of blood culture contamination (BCC), blood culture (BC) needs to be repeated. This may delay appropriate treatment, prolong hospitalization and, consequently, increase its costs. The aim of the study was to assess the frequency of BCC and associated factors in a general hospital in Poland based on reports of BC in samples submitted for laboratory testing in 2019–2020. BCC is recognized when bacteria (especially those belonging to natural human microbiota) are isolated from a single sample and no clinical signs indicated infection. True positive BC is confirmed by the growth of bacteria in more than one set of blood samples with the corresponding clinical signs present. The structure of BC sets, microorganisms, and laboratory costs of BCC were analyzed. Out of 2274 total BC cases, 11.5% were true positive BC and 9.5% were BCC. Of all the BCC identified in the entire hospital, 72% was from Internal Medicine (IM) and Intensive Care Unit (ICU) combined. When single sets for BC were used in IM in 2020, the use increased to 85% compared with 2019 (*p* < 0.05). The predominant isolates were coagulase-negative staphylococci (84%). The estimated extra laboratory costs of BCC reached almost EUR 14,000. The BCC was a more serious problem than expected, including non-recommended using of single BC sets. Compliance with the BC collection procedure should be increased in order to reduce BCC and thus extra hospital costs.

## 1. Introduction

One of the key tools available to clinicians for differentiating clinical presentations of bloodstream infections (BSIs) in patients is microbiological diagnosis. Blood culture (BC) can be used to confirm infectious etiology, isolate etiologic agents, and determine their drug susceptibility, and can form the basis for the implementation of targeted therapy [1,2,3].

The use of less effective empirical therapy, including broad-spectrum antibiotics, places greater financial burdens on the healthcare provider and can increase drug resistance [4]. Furthermore, targeting the specific pathogen and employing focused therapy can significantly improve the final outcome [5]. Delayed appropriate treatment may result in prolonged hospitalization, inappropriate antibiotic therapy, and consequently greater exposure to drug side effects and toxicity [4,5,6].

In clinical practice, false positive cultures are sometimes reported following contamination with human microbiota; these are known as blood culture contaminations (BCCs). However, distinguishing true BSI from contamination remains a challenge for physicians and microbiologists [7,8]. The contamination rate can be calculated in two methods. The blood culture contamination rate (%) was calculated according to the following formula: (1) the number of BCC × 100% to the number of total positive BC (true and false positive BC), (2) the number of BCC × 100% to the number of total BC (true positive BC, false positive BC, true negative BC, false negative BC). Despite the development of culture techniques and automation of the identification process, some laboratories report BCC rates of nearly 50% of total positive BC [7,8]. Clinical and Laboratory Standards Institute recommendations indicate that contamination rates for adults in health care settings should not exceed 3%, measured as the total number of blood cultures (total BC) [9,10]. Some authors record BCC rates ranging from 0.6% to 6%, which only emphasizes the magnitude of the problem [11,12]. False positive BC events increase the cost of additional laboratory tests, by up to 20%, cause unwarranted antibiotic prescription, and are associated with a 40% increase in antimicrobial spending. BCC also results in prolonged hospitalization, i.e., of up to 5 days [9,10,11,12,13]. Studies in the United States estimate the hospital costs associated with BCC to range from USD 2844 to USD 10,078 [8,13].

In order to effectively prevent the consequences of BCC, it is first necessary to understand and eliminate its causes. In addition, for individual health care units, there is a need to identify the key critical issues.

Standard precautions must be taken during the sampling process, aseptic conditions must be rigorously maintained, and all rules included in the procedures must be strictly followed. One of the most important causes of BCC is the use of improper techniques for collecting material for laboratory testing, e.g., incorrect use of protective gloves, improper preparation of media bottles, or taking samples in an order that does not conform to the protocol [13,14,15,16,17].

It is equally important that the injection site is correctly disinfected when taking samples, e.g., the preparation must be left on the skin long enough and the disinfected site should not be touched. Failure to follow these basic rules results in the transfer of bacteria physiologically present on the skin to the culture medium bottles and a subsequent false positive result [16,17].

The results of a number of clinical laboratory investigations indicated that certain microorganisms are more likely to contaminate blood samples. These include coagulase-negative staphylococci (CoNS), including *Staphylococcus epidermidis*, most *Corynebacterium* species, *Bacillus* spp., *Propionibacterium* spp., *Micrococcus* spp., and *Cutibacterium acnes* [9,16,18,19]. These microorganisms are commonly found on the skin, and although they can cause serious infections under certain conditions, their detection in a single BC is considered a contaminant without clinical significance [16,20].

To help differentiate between contamination and true bacteremia, a sufficient number of BC diagnostic sets should be collected; a set is defined as two bottles of culture medium: one aerobic and one anaerobic. It is also generally recommended that two- or three-bottle sets are used; the sensitivity of a single blood culture set is limited due to the periodic appearance of bacteria and fungi in the bloodstream [21]. A study by Lee et al. found that BC sensitivity increased when more than one blood set was used; in addition, contamination usually occurs in a single bottle from a set, while cases of BSI demonstrate positivity in several samples [22]. Another reason for taking cultures in multiple sets, from anatomically different sites, is that while contamination usually occurs in a single bottle from a set, cases of BSI demonstrate positivity in several samples [22,23].

There is a large gap in the current knowledge regarding the problem with BCC in hospitals in Poland. As such, the aim of the study was to evaluate its incidence in a general hospital. The study itself included the following critical data for clinically insignificant results: time of sampling (month, day of the week, a shift/day-night), a quantitative-qualitative analysis of the microorganisms isolated from BCC, the choice of the number of diagnostic sets used, and the costs resulting from the contamination.

## 2. Materials and Methods

### 2.1. Study Design and Data Collection

The study consisted of a retrospective analysis of BC results collected by a microbiology laboratory between 1 January 2019 to 31 December 2020. The study was conducted in a general hospital (16 departments; 380 beds) (Subcarpathian Voivodeship, Tarnobrzeg, Poland). The data collection process, including the conduct of microbiological tests, criteria for diagnosing BSI and BCC and the report-generation procedure, were standardized according to the laboratory procedures.

A total of 2274 blood cultures were analyzed. True positive BC was defined as a case in which a microorganism potentially representing an etiological agent of BSI was cultured, consistent with the patient’s clinical condition. A laboratory result found to be true positive was labeled with the alert microorganism. To assess the clinical significance of organisms that are often considered contaminants when isolated from blood cultures (coagulase-negative staphylococci, *Corynebacterium* spp., *Bacillus* spp., and *Propionibacterium* spp.), the clinical microbiology laboratory used an algorithm [21]. If there is a suspicion of BCC, an additional sample should be collected within 48 h, after a microbiologist reports the first sample as probably contaminated. When no additional blood cultures are obtained from a patient within 48 h, a microbiologist discusses the potential significance of the isolate with a physician caring for the patient. On the basis of the assessment clinical history, disease symptoms, and additional laboratory tests, the isolate was classified as a pathogen or as a contaminant.

As a number of single BC sets were sent for microbiological analysis, despite recommendations, any single positive blood sample in which natural microbiota isolated was identified for consultation with the main doctor. If no clinical markers reflecting infection were present, the culture was considered as BCC. As only single BC sets were investigated in many cases, this contact with the main doctor was crucial to establish whether the other results confirm the infection. The absence of pathogen growth in the collected specimen indicated a negative culture [24].

### 2.2. Methods

#### 2.2.1. Blood Culture Technique

Blood for culture was collected on liquid media in BACTEC bottles: AERO (aerobic culture), ANAERO (anaerobic conditions), or PEDS (pediatric, dedicated for children), and then incubated in a BD BACTEC 9240/9120 (Becton, Dickinson and Company, Franklin Lakes, NJ, USA, 2012) for 5 days. The instrument detects any increase in the level of carbon dioxide produced by the microorganisms, typically identifying a positive sample during the first 2 or 3 days of incubation. In the next step, a gram-stained direct preparation was made to provide initial information about the microorganisms. The culture from positive blood cultures was streaked onto appropriate solid media. From the BACTEC AERO bottle, the specimen was inoculated onto COS (Columbia agar with Sheep Blood) and CPS (Chromagar Orientation) (BioMérieux, Marcy-l’Étoile, France) media and incubated at 35 ± 2 °C for 18–24 h under aerobic conditions. From a BACTEC ANAERO bottle, blood samples were cultured on the SCS (Schaedler Agar +5% Sheep Blood) and SNVS (Schaedler Neo. Vanco. Agar +5% Sheep Blood) (BioMérieux) medium; these were incubated under anaerobic conditions at 35 ± 2 °C for 18–24 h. The cultures on COS and CPS media were also performed under the same conditions as for aerobic bottles. At the end of the incubation, the morphology (colony structure) and type of culture (aerobic, anaerobic) were determined, the cultured microorganisms were identified, and their drug susceptibility evaluated according to 2019 and 2020 EUCAST recommendations with the Vitek system (BioMérieux) [25].

#### 2.2.2. Microbiological Reports

The results of blood tests and annual statements with details of hospital departments were obtained using a laboratory information system (LIS Centrum MARCEL S.A., Zielonka, Poland). The reports provided data on, inter alia, the number of Total BC, the number of BCC, the number of true positive BC, the number of negative BC, the number of blood culture set, the date and time of specimen collection for testing.

Based on these findings, the following variables were selected: month, day of the week, and work shift (day-night); in the case of the latter, day shift was assumed to indicate on-call duty from 7:00 a.m. to 7:00 p.m. and night shift on-call duty from 7:00 p.m. to 7:00 a.m. The week was divided into working days (Monday–Friday) and weekends (Saturday–Sunday). The laboratory results allowed for a qualitative and quantitative summary of the isolated microorganisms.

In further parts of the analysis, relating to the critical points of BCC occurrence, the frequency of BCC was presented as the rate of BCC with respect to all positive BC samples (true and false) according to international standards [10].

This study was reviewed and approved by the laboratory manager.

#### 2.2.3. Analysis of Laboratory Costs of Contamination

The costs incurred by contamination were determined by calculating the additional materials needed to repeat the microbiological testing of BC by the laboratory. The analysis was performed using Excel (Microsoft Office 2019, Microsoft Corporation, Redmond, WA, USA). The cost simulation was performed for all hospital departments that had BCC in their microbiology reports. The calculation took into account the number of BCC generated by a given department, and the costs of used BC sets and materials (i.e., media, reagents, and antibiotics needed to perform an antibiogram). The analysis assumed that retesting is performed according to standard procedures, that is, using at least two sets of transport media (except for testing in neonates and children with body mass < 36.3 kg) [24]. It was not possible to estimate the costs of treating patients in the departments: no permission was provided to collect the relevant data.

### 2.3. Statistical Analysis

The statistical analysis was performed using Statistica version 13.1 CSS software (StatSoft Polska Sp. z o.o., Kraków, Poland). A One-way analysis of variance (ANOVA) with the post hoc Fisher’s LSD test was applied to classify departments by mean monthly BCC (BCC m-index), a Friedman ANOVA test was applied to evaluate the profiles of the blood culture collection sets used in the classified departments. BCC m-index was calculated using the formula BCC m-index = N_BCC per month_/N_Total BC,_ where N = the number of BCC in the month, and N_Total BC_ = the number of all blood cultures in over 2 investigated years. The Wilcoxon matched pairs test was used to compare the rate of BCC across months and days of the week, and the Chi-square test on day and night work shifts, and on working days (Monday–Friday) and the weekend (Saturday–Sunday). The level for statistical significance was set at *p* < 0.05.

## 3. Results

### 3.1. Characteristics of Blood Culture and Contamination throughout the Hospital

Of all the BC samples received (2274) in the microbiology laboratory during the study period (2019–2020), 479 (21.06%) were positive BC samples, including both real BC, i.e., true positive; and BCC, i.e., false positive. The data indicate that 217 (9.54% of Total BC) samples appeared contaminated (8.81% in 2019 and 10.62% in 2020), while 262 (11.52%) had actual bacteremia (being true positives), as shown in Table 1.

The majority of sets retrieved for testing were single sets (88.08%) in both 2019 (87.19%) and 2020 (89.38%) (Table 1).

#### 3.1.1. Percentage of BCC in Hospital

**Figure 1:**
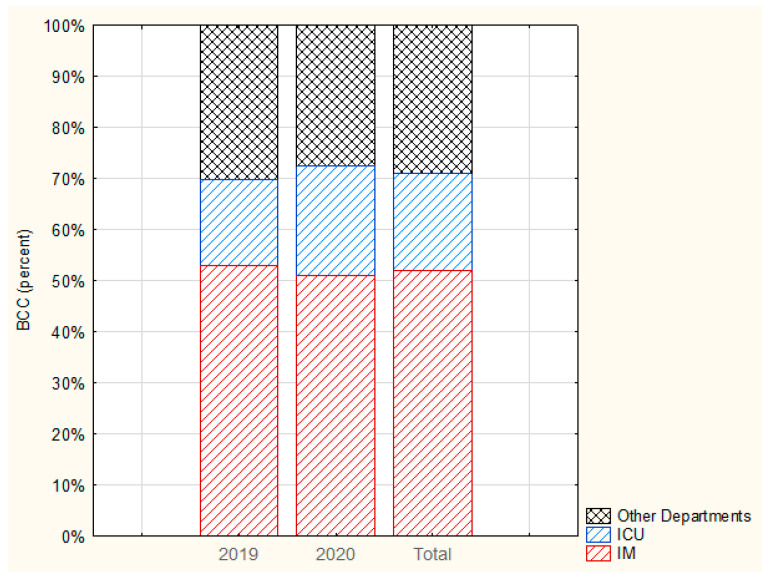
Percentage of BCC in IM, ICU and Other Departments in 2019 and 2020.

During the 2-year follow-up period, the departments, Internal Medicine (IM), Intensive Care Unit (ICU), Oncology, Infant Care, and Neurology, collected the highest number of BC samples compared with other departments (>130 samples), while also having a noticeably high number of BCC (>10). The IM and ICU departments together accounted for 71.89% of BCC in the entire hospital (IM: 52.07%; ICU: 19.82%) (Figure 1).

#### 3.1.2. Mean Monthly BCC Index in Hospital

**Figure 2:**
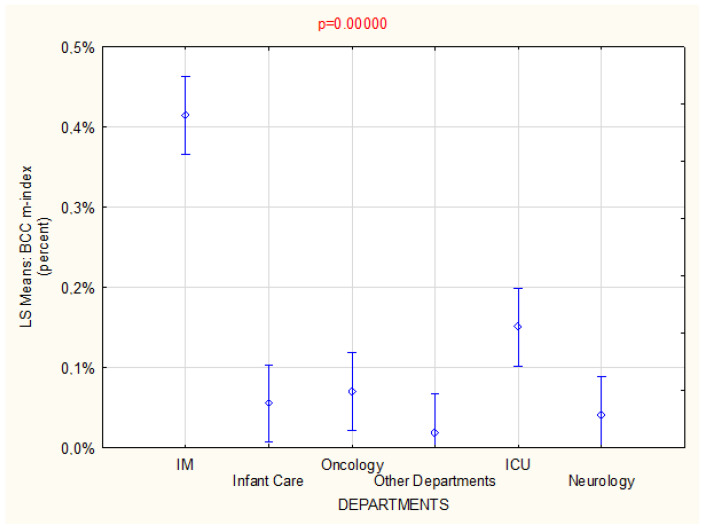
Characteristics of BCC m-index in hospital departments for the period of 2019–2020.

The IM and ICU departments demonstrated a significantly higher level of sample contamination compared with other departments. (*p* < 0.02), indicted by the distribution of BCC samples in relation to all tests performed in over 2 years. As no statistically significant differences were observed between the other departments in this regard (*p* > 0.05), they were not presented in detail (Figure 2).

#### 3.1.3. Number of BCC and Total BC Samples in Hospital

Among the analyzed departments, some had no contamination (Pediatrics, Orthopedics, Infectious Disease, Surgery); however, these departments collected relatively few culture samples (Table 2).

#### 3.1.4. Structure of Contaminant Species in Hospital

**Figure 3:**
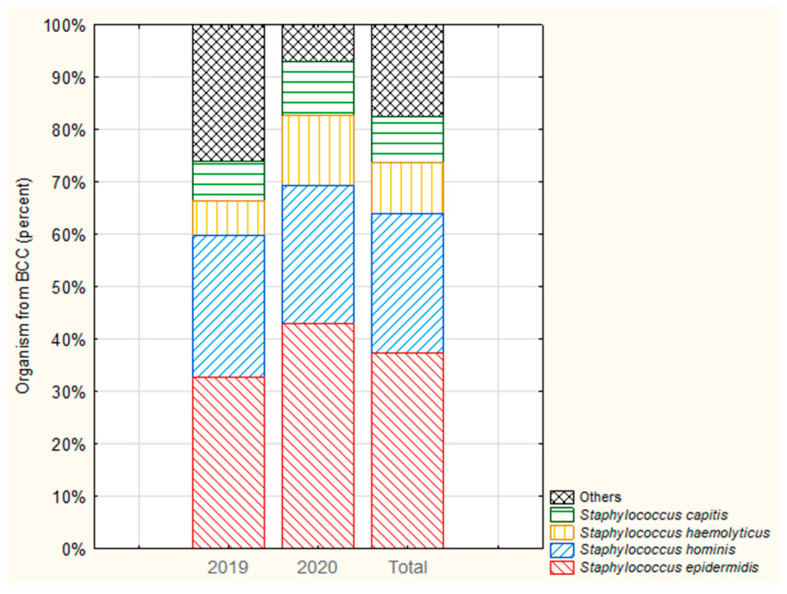
Distribution of the four most prevalent organisms isolated from blood culture contamination (BCC) in 2019 and 2020.

During the study period, 20 pathogens were found to be responsible for BCC, these being (from most frequently isolated to the least): *S. epidermidis*, *S. hominis*, *S. haemolyticus*, *S. capitis*, *Kocuria* spp., *S. warneri*, *S. auricularis*, *Micrococcus* spp., *Achromobacter xylosoxidans*, *Bacillus species*, *Granulicatella adiacens*, *Lactococcus garvieae*, *Pediococcus pentosaceus*, *Prevotella disiens*, *Propionibacterium acnes*, *S. caprae*, *S. cohnii*, *S. pseudintermedius*, *Streptococcus mitis*, and *Streptococcus salivarius*. The most commonly isolated pathogen in BCC samples was *Staphylococcus epidermidis* (37.33%), which together with three other coagulase-negative staphylococci (*S. hominis*, *S. haemolyticus*, and *S. capitis*) accounted for 84.3% of BCC (according to the rank selection) relative to all hospital-wide BCC (Figure 3).

### 3.2. BCC vs. Critical Points for Obtaining a Clinically Insignificant BC Result

The following analyses refer to the IM and ICU departments; these two most frequently collected BC for testing and were hence the main BCC contributors in the entire hospital. Two approaches were used to present the data: (1) for two departments combined (“IM + ICU”) as a cumulative source of BCC in a hospital, and (2) data were collected separately for IM and ICU (2-year perspective or comparing 2019 to 2020).

#### 3.2.1. BCC by Month and Day of the Week

**Figure 4:**
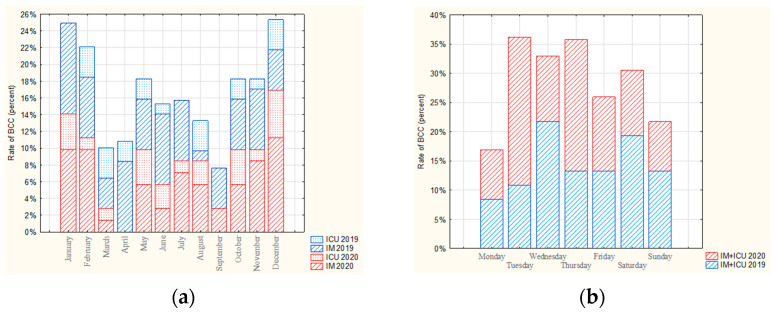
The rate of BCC for IM and ICU in 2019 and 2020 by: (**a**) month, (**b**) day of the week.

Contamination rates for the two departments combined (IM and ICU) varied from month to month over the 2-year study period; however, no significant difference in BCC rate was found between months (*p* > 0.05). In 2019, the highest BCC rates were observed in January, February, and April (10.8% in each) and the lowest in August and September (4.8% in both). In 2020, the highest BCC rate was recorded in December (17.35%), while no contaminated samples were identified in April (Figure 4a).

The BCC rate for the total number of contaminations in the IM and ICU units according to weekday differed between 2019 and 2020. In 2019, the highest rate of BCC was recorded for Wednesday (21.7%) and the lowest for Monday (8.4%), while in 2020, the highest BCC was observed on Tuesday (25.4%) and the lowest on Monday (8.5%) (Figure 4b). However, no statistically significant difference in contamination rate was observed between different weekdays (*p* > 0.05).

**Figure 5:**
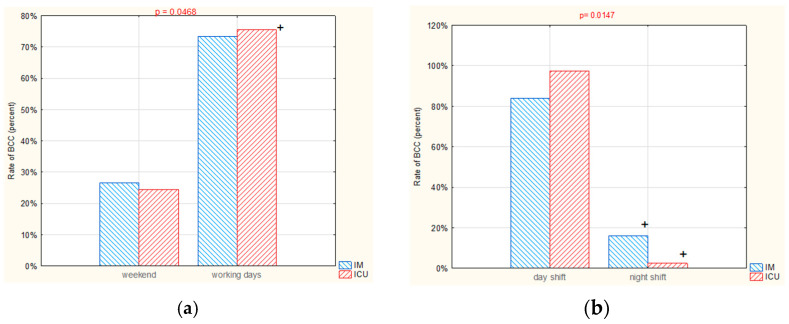
Rate of BCC on IM and ICU in 2019–2020 according to: (**a**) part of the week (working days vs. weekend), (**b**) type of shift (day vs. night). +: *p* < 0.05.

Over the whole 2-year period, a higher BCC rate was observed in each of the departments during the working week (Monday–Friday), even when taking into account the different number of days per part of the week. However, this difference was only significant for the ICU (*p* < 0.05), with rates of 75.6% in the week and 24.4% in the weekend (Figure 5a).

#### 3.2.2. BCC by Shift (Day vs. Night)

The rate of BCC in both the IM and ICU differed between the night shift and the day shift during the 2-year period. As suspected, each department generated more BCCs on the day shift than on the night shift (IM: 82.3% vs. 17.7%, ICU: 97.6% vs. 2.4%, day vs. night respectively; *p* < 0.05) (Figure 5b).

#### 3.2.3. Relation of Number of BC Sets to BCC Frequency

**Figure 6:**
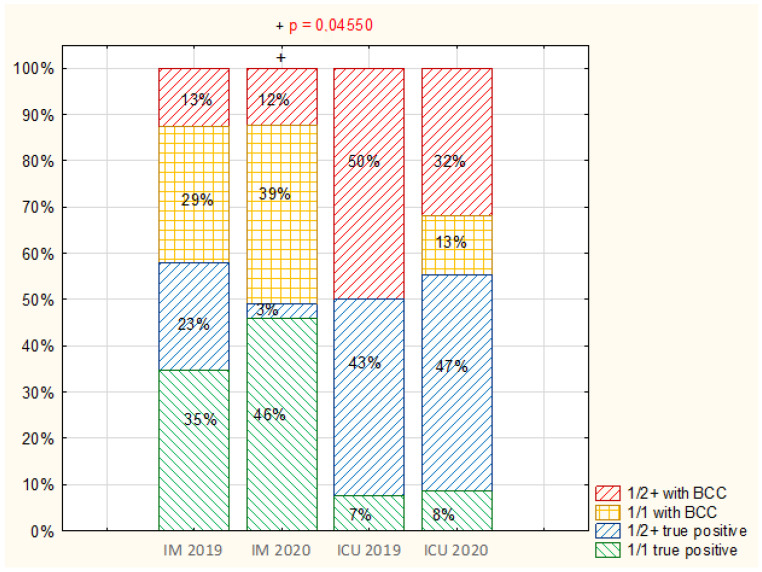
Comparison of the percentage of single (1/1) and the percentage of at least double (1/2+) sets with BCC and with infection (true positive) (analysis without negative samples) in the Internal Medicine (IM) department and Intensive Care Unit (ICU) in 2019 and 2020.

In the IM department, in 2019, single sets (1/1) were used frequently (64%) for routine diagnosis, and nearly 30% of these single sets demonstrated BCC. Even more single sets were used in 2020 (84.7%), and the proportion of these sets with BCC was nearly 10% higher than in the previous year (Figure 6) (*p* < 0.05).

In contrast, ICUs in 2019 mainly used double, or more than two (1/2+), blood culture sets (92.5%); additionally, no contamination was found in single sets, but at least double sets with BCC (1/2+) accounted for 50% of all sets. In 2020, BCC was recorded in nearly 13% of single (1/1) sets; however, no significant, change was observed in the profile of BC sets used, compared with 2019 (Figure 6) (*p* > 0.05).

#### 3.2.4. BCC by Organism

**Figure 7:**
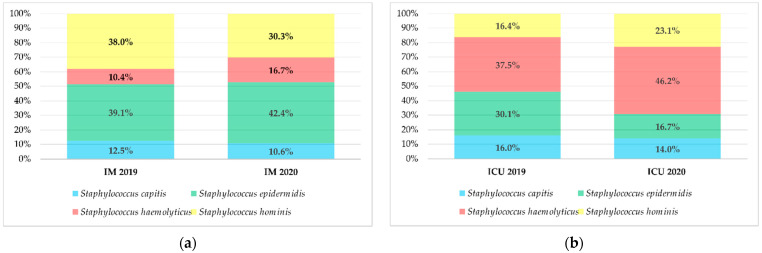
Distribution of the four major species of blood culture contaminants in 2019 and 2020 in the: (**a**) Internal Medicine (IM) department, (**b**) and Intensive Care Unit (ICU).

In both departments, the most commonly isolated microorganisms from contamination in both 2019 and 2020 were coagulase-negative staphylococci. In 2019, the most prevalent species isolated from BCC on the IM department was *S. epidermidis* (39.1%); in 2020, it was also *S. epidermidis* (42.4%) (Figure 7a). In the ICU, *S. haemolyticus* was most commonly isolated in BCC samples: 37.5% and 46.2% (in 2019 and 2020 respectively) (Figure 7b).

### 3.3. Laboratory Costs Due to Contamination (Analysis for All Hospital Departments)

The additional financial burden of contamination for the entire hospital was 61,229 PLN over 2 years. Taking into account the average euro exchange rate provided by the National Bank of Poland for 2019 and 2020, respectively, it was calculated that the total laboratory cost of BCC for the 2 years was EUR 13,986.87.

The highest costs were generated by the IM and ICU departments, together accounting for nearly 75% of the total laboratory costs related to BCC (i.e., 45 353 PLN) (Table 3). The costs due to contamination increased from 28,427 PLN in 2019 to 32,802 PLN in 2020.

## 4. Discussion

Very few studies have attempted to assess the true scale of the problem of BCC, and none have made a simultaneous assessment of the possible financial consequences of sample contamination. Our present findings indicate that a high rate of BCC is not only a serious financial problem and a challenge for hospital managers, but also a potentially serious threat to the health and life of the patient. Identifying the source of BCC is very important in the diagnosis and management of patients with a suspicion of BSI. Controlling the frequency and sources of contamination, and taking steps to reduce it, can also minimize the unwarranted prescription of antibiotic therapy and the associated consequences of increasing drug resistance and adverse side effects. An analysis of BC results is a valuable source of information about the epidemiological status of a hospital [21,26]. It also makes it possible to assess compliance with microbiological sampling procedures.

Our findings indicate that the incidence of BCC for the entire hospital was 8.81% in 2019 and 10.62% in 2020. These results were much higher than the recommended target rate of 3% for BCC in adults [10]. Lalezari et al. report that 50% of all positive blood cultures were found to contain contaminants [27]. Washer et al. found 13% of all positive blood cultures appeared to be contaminated, and that overall blood contamination rates were 0.8% among cultures obtained peripherally by phlebotomists [19]. Rupp et al. found contamination in 23% of all positive blood cultures, and that overall contamination rates were 1.8% during a defined study period [11]. Story-Roller and Weinstein found contamination in 26% of all positive blood cultures and the overall contamination rate to be 3.9% [28].

It can be seen that the scope and magnitude of blood culture contamination is influenced by a range of factors. Efforts to reduce the rate of BCC should begin with identifying the departments that generate the most BC contamination. Targeting busy departments with high rates of BCC, such as IM and ICU, and implementing specific measures as a part of quality improvement interventions, will effectively reduce the rate of culture contamination [14]. The scale of this problem is also underscored by the fact that the rate of BCC observed in the study was close to true positive BC (11.70% in 2019 and 10.62% in 2020).

The bacteria of the natural physiological microbiota of patients and staff can be transferred to transport media during venipuncture, and this contamination can be diagnosed as BCC. The most common bacteria isolated from BCC were coagulase-negative staphylococci (CoNS) (84.3%), followed by other skin contaminants; this is in line with the results of similar studies [8,9,13]. For example, Weinstein et al. isolated a number of organisms representing contamination from adult patients in three hospitals around the USA: CoNS, *Corynebacterium species*, *Bacillus species* other than *Bacillus anthracis*, *Propionibacterium acnes*, *Micrococcus* species, viridans group streptococci, enterococci [29,30]. Our present data indicate that *Staphylococcus epidermidis* was the most frequent contaminant, which is consistent with the results in other papers [8,14]. According to the laboratory procedures, every contaminated BC result was consulted with a doctor, regardless of the number of blood cultures set.

A key aim of the study was to identify the key critical issues that may result in a higher incidence of BCC. Hence, the time of sampling was regarded as a critical point, more specifically month, day of the week and day/night shift. Regarding the variation across months during the 2-year follow-up period, no variation in BCC level was observed. This is in line with Hemeg et al., who also do not report any significant difference in contamination rates between months over a span of 1 year [8]. In contrast, Min et al. reported significant differences in contamination rates between months, with the highest rates seen in August and November and the lowest in February [31]. It can only be assumed that the number of samples from BCC will be higher in the summer months (vacation season), when there are staff shortages and the hospital is supported by external staff not permanently employed at the facility. However, no studies, if any, have examined whether the BCC rate is higher in any particular month during the year or on any days during the work week.

When the week was divided into workdays and weekends, only the ICU demonstrated a higher rate of BCC during workdays compared with weekends (more routine duties, pace of work). We suspected that the rate of BCC will be the highest on Monday due to the heavy workload after the weekend; however, no significant difference was observed. In addition, no statistically significant difference in contamination rate was found between different weekdays; this can be caused by the multitude of factors associated with the collection procedure, e.g., personal factors, lack of trained personnel, and employee rotation. The influence of the human factor (number of specialized personnel, age, seniority, checking knowledge of the procedures) should be taken into account in further studies. It can be assumed that a higher number of specialized personnel can contribute to a decrease in the number of incorrect BC collections.

Our study is one of the few to evaluate whether the BCC rate differs between day and night work shifts. The departments with the highest numbers of contaminations also demonstrated significantly more frequent clinically insignificant microbiological test results in blood during the day shift. This was likely related to the increased workload during the day shift, i.e., more patients to be cared for and more admissions to the department, as well as too few employees and employee rotations, which may have led to inadequate adherence to BC collection procedures. However, Jusoh et al. report a significantly higher risk of BCC during night shift work in the emergency department: BC collected during the night shift was almost five times more likely to be contaminated than that collected during the morning shift [32].

In the present study, our profile analysis found that the IM department used mainly single sets for collecting blood for cultures. As a result, the patients in this department were more likely to obtain a BCC result than those in the ICU department, and hence require another examination and a longer hospital stay, i.e., by 5 days on average [9,13]. This problem was most likely observed because of an insufficient understanding of the blood sampling procedures by staff on clinical departments. The time needed to repeat the test may play a role, as well as the necessity to perform other laboratory tests that may be helpful in diagnosing BSI, e.g., determination of C-reactive protein or procalcitonin levels. Any delay in making the correct diagnosis carries certain consequences [33]. Ferrer et al. found a delay in the administration of the first targeted antibiotic due to improperly collected specimens to be significantly related to an increase in in-hospital mortality in patients with severe sepsis and septic shock [6].

Since bacteria and fungi may not be constantly present in the bloodstream during infection, the sensitivity of a single blood culture set is very limited. In our study, 9.5% of the single blood culture sets collected at the IM and ICU departments were true positive, while 44.83% of double sets were true positive. For samples with more than two sets, the percentage was 29% (data not shown in table). However, no attempt was made to determine the cumulative sensitivity of blood cultures in the present study. Studies performed in the 1970s, 1980s, and early 1990s found that performing two to three blood cultures from samples obtained from adults during a 24-h period could detect 99% of all BSIs [22,33,34,35,36,37,38]. Cockerill et al. reported that two blood cultures detected only 80% of BSIs, three detected 96% of BSIs, and four were required to detect 100% of BSIs; however, these results were unexpected given the use of a modern CMBCS (continuous-monitoring blood culture systems) and contemporary culture media. The authors suggest that newer systems may detect bacteremia at lower levels than older systems and that higher numbers of blood cultures should be used to detect low-level bacteremia [36]. Similar results were obtained by Lee at al., in an evaluation of the cumulative sensitivity of BC collected sequentially over 24 h using monitored automated systems; it was found that the cumulative yield of pathogens from three blood culture sets (two bottles per set), with a blood volume of 20 mL in each set (10 mL per bottle), was 73.2% for the first set, 93.9% for two sets, and 96.9% for three sets. To achieve a detection rate of >99%, as many as four blood cultures may be needed [22].

Unlike previous studies, our present paper also evaluates the additional laboratory costs caused by contamination in BC samples; however, this value is only a component part of the total additional cost associated with BCC. The overall structure of additional costs should take into account the costs of medications used, including antibiotics that were prescribed due to a clinically irrelevant result, the prolonged diagnostic process, and those associated with prolonged hospitalization, including costs related to waiting time for a repeat result of a microbiological blood test or other ancillary tests. Unfortunately, no detailed data on the amount of costs listed were available, and this constitutes a limitation to our present analysis. Gander et al. reported a difference in median patient charges between negative and false positive episodes of USD 8720 per contamination (a 47% increase); however, in this study, laboratory costs were not separated from the other expenses [18].

In contrast, Alnami et al. found a four-fold increase in microbiology charges per BCC compared with a negative BC [14]. In the latter case, the calculation takes into account the fact that an initial positive culture result does not yet indicate whether the result is true or false; therefore, each retest and subsequent establishment of the antimicrobial susceptibility profile involves further costs. These costs can be avoided for the negative result, provided it is not contaminated. Thus, even if the result ultimately turns out to be clinically insignificant (false positive, BCC), this does not remove the need to perform the next step of the test according to the procedure (real cost of BCC), as the first step could not rule out the possibility of a true positive result (infection).

The study has some limitations. Firstly, the number of BC samples was limited due to a change in the IT system in the hospital laboratory, resulting in a loss of data prior to 2019. In addition, many single sets of BC may be not fully informative. Finally, the study is retrospective in nature (stagnant data), thus yielding less data about the clinical status of the patient. However, the key strength of the study is its comprehensiveness, performing a simultaneous epidemiological, microbiological, and economic analysis associated with the occurrence of BCC.

## 5. Conclusions

The BCC rates for the hospital significantly exceeded internationally acceptable levels; the IM and ICU departments ordered the most collections, generating the highest number of contaminations. In order to reduce the rate of BCC and thus lower extra hospital costs, greater adherence to the BC collection procedure should be encouraged, e.g., correct disinfection of skin, and strictly adhering to the rule of using at least two BC sets. Although the study is limited by the use of single blood culture sets, the rate of BCC is very high. Our findings underline the need for greater staff training in this regard and much better communication between the laboratory and the hospital departments. Further prospective studies are needed to identify personal and organizational factors related to higher rates of BCC.

## Figures and Tables

**Table 1:** General characteristics of blood cultures performed at the hospital in 2019–2020.

Variable		2019	2020	Total
Total BC		1351	923	2274
True positive BC, n (%)		162 (11.99)	100 (10.83)	262 (11.52)
True negative BC, n (%)		1071 (79.27)	725 (78.55)	1796 (78.98)
BCC, n (%)		119 (8.81)	98 (10.62)	217 (9.54)
Rate of BCC (%)		42.35	49.49	45.30
Sets retrieved, n (%)	1	1178 (87.19)	825 (89.38)	2003 (88.08)
	≥2	173 (12.81)	98 (10.62)	271 (11.92)

BC—Blood Culture, BCC—Blood Culture Contamination, Rate of BCC—number of BCC to true positive BC.

**Table 2:** Number of BCC versus total BC samples among various hospital departments in 2019 and 2020.

Department	BCC			Total BC		
	2019	2020	Total	2019	2020	Total
Internal Medicine	63	50	113	666	471	1137
ICU	20	21	41	69	77	146
Oncology	13	6	19	81	55	136
Infant Care	9	6	15	209	138	347
Neurology	2	9	11	79	66	145
Cardiology	4	2	6	58	26	84
Infectious Disease	6	1	7	33	17	50
Others	2	3	5	156	73	229

BC—Blood Culture, BCC—Blood Culture Contamination, ICU—Intensive Care Unit.

**Table 3:** Hospital laboratory cost profile due to occurrence of blood culture contamination in 2019 and 2020.

Department	2019 Costs of BCC [PLN]		2020 Costs of BCC [PLN]		Total Cost of BCC [PLN]
	Real	Extra	Real	Extra	
Internal Medicine	7021	5428	9702	7700	29,851
ICU	4974	4214	3080	3234	15,502
Oncology	1824	599	3080	924	6427
Infant Care	783	522	693	462	2460
Neurology	251	1091	308	1386	3036
Cardiology	425	251	616	308	1600
Infectious Disease	522	87	462	77	1148
Others	174	261	308	462	1205
Total	15,974	12,453	18,249	14,553	61,229

BCC—Blood Culture Contamination, ICU—Intensive Care Unit.

## Data Availability

The data presented in this study are available on request from the corresponding author.
